# The Role of MPK6 as Mediator of Ethylene/Jasmonic Acid Signaling in *Serendipita indica*-Colonized Arabidopsis Roots

**DOI:** 10.1007/s11105-018-1077-z

**Published:** 2018-03-28

**Authors:** R. Daneshkhah, F. M. W. Grundler, Krzysztof Wieczorek

**Affiliations:** 1 Division of Plant Protection, Department of Crop Sciences, University of Natural Resources and Life Sciences, Konrad-Lorenz-Straße 24, 3430 Tulln on the Danube, Austria; 2 Institute of Crop Science and Resource Conservation, Molecular Phytomedicine, University Bonn, Karlrobert-Kreiten-Str. 13, 53115 Bonn, Germany

**Keywords:** *Serendipita indica*, *mpk6*, Hormone signaling, Ethylene, Jasmonic acid

## Abstract

*Serendipita indica* is an axenically cultivable fungus, which colonizes a broad range of plant species including the model plant *Arabidopsis thaliana*. Root colonization by this endophyte leads to enhanced plant fitness and performance and promotes resistance against different biotic and abiotic stresses. The involvement of MPK6 in this mutualistic interaction had been previously shown with an *mpk6 A. thaliana* mutant, which failed to respond to *S. indica* colonization. Here, we demonstrate that *mpk6* roots are significantly less colonized by *S. indica* compared to wild-type roots and the foliar application of plant hormones, ethylene, or jasmonic acid, restores the colonization rate at least to the wild-type level. Further, hormone-treated *mpk6* plants show typical *S. indica*-induced growth promotion effects. Moreover, expression levels of several genes related to plant defense and hormone signaling are significantly changed at different colonization phases. Our results demonstrate that the successful root colonization by *S. indica* depends on efficient suppression of plant immune responses. In *A. thaliana*, this process relies on intact hormone signaling in which MPK6 seems to play a pivotal role.

## Introduction

*Serendipita indica* (former *Piriformospora indica*), a mutualistic biotrophically living fungal endophyte from the order Sebacinales (Weiss et al. 2004), colonizes plant roots transferring various benefits to the host including growth promotion, increase in yield as well as abiotic and biotic stress tolerance (reviewed in Qiang et al. 2012). The fungus colonizes root tissue very effectively in four phases: (1) extracellular (approx. 1 dpi); (2) biotrophic (less than 3 dpi); (3) cell death associated (approx. 7 dpi); and (4) fungal reproduction phase (approx. 14 dpi). During development, the fungus triggers increased nitrate and phosphate uptake as well as boosted plant metabolism (Yadav et al. 2010). In return, *S. indica* receives carbohydrates from the plant (Schäfer et al. 2009). Some plant components have been identified, which are known to be required during the *S. indica*-plant interaction leading to fungus*-*induced growth promotion. Among these, an atypical receptor kinase with leucine-rich repeats (Shahollari et al. 2007) and a serine/threonine kinase (Camehl et al. 2011) required for the full activation of two mitogen-activating protein kinases 3 and 6 (MPK3 and MPK6) have been identified. In *A. thaliana* MPK6 is involved in various aspects of developmental processes and stress responses in which plant hormones such as jasmonic acid (JA) and ethylene (ET) are intertwined (Bethke et al. 2009). Vadassery et al. (2009) found that an *mpk6* knock-out mutant failed to respond to growth promotion effects triggered by *S. indica*. The authors observed that activation of MPK6 after application of the cell wall extract from *S. indica* to the roots was even stronger than its activation triggered by bacterial flg22. These results suggest that MPK6 and its induction via Ca^2+^ play an important role in this mutualistic relationship (Vadassery et al. 2009). Further, the phytohormones ET and JA as well as their operative signaling pathways are involved in regulation of *S. indica* colonization and exert the beneficial effects triggered by the mutualist (Jacobs et al. 2011; Khatabi et al. 2012). This is supported by the fact that mutants, which are impaired in JA biosynthesis or signaling, show elevated root immune responses. This leads to reduced *S. indica* root colonization indicating that JA might regulate early immune responses by suppression of related defense pathways (Jacobs et al. 2011). In fact, several recent reports showed that the successful root colonization is accompanied by down-regulation of plant defense responses (Schäfer et al. 2009; Camehl et al. 2011; Jacobs et al. 2011; Vahabi et al. 2015). Further ET biosynthesis, signaling, and ET-targeted transcription factors are required for colonization and the beneficial effects of *S. indica* in barley and *A. thaliana* (Camehl et al. 2010; Khatabi et al. 2012). Summarizing all these evidences, it seems that MPK6 through proper ET/JA signaling might play central and pivotal role in mediating beneficial effects of *S. indica* to the host plant.

Here, we demonstrate that JA/ET signaling pathways through the MPK6 cascade are crucial in the establishment of proper *S. indica* root colonization and induction of its beneficial effects in *A. thaliana*. As previously shown (Vadassery et al. 2009), *mpk6* mutant does not exhibit the typical growth phenotype when colonized by *S. indica*. We demonstrate that this is due to defective fungal root colonization, which is a result of a compromised hormone signaling in these plants. Our results show that the *mpk6* is able to respond normally to the fungus after systemic application of phytohormones. These treatments restore root colonization to a level similar to that in wild type roots. Subsequently, *S. indica* induces normal growth promotion effects. To explore the molecular mechanisms behind this phenomenon, we investigated the expression of genes involved in hormone- or defense-related pathways in both wild type and *mpk6*. Our results substantiate the role of MPK6 in the essential molecular steps leading to *S. indica*-induced growth promotion in *A. thaliana*.

## Results

### Systemic Application of Eth or mJA Retrieves Normal Growth Promotion Triggered by *S. indica* in *mpk6*

**Fig. 1:**
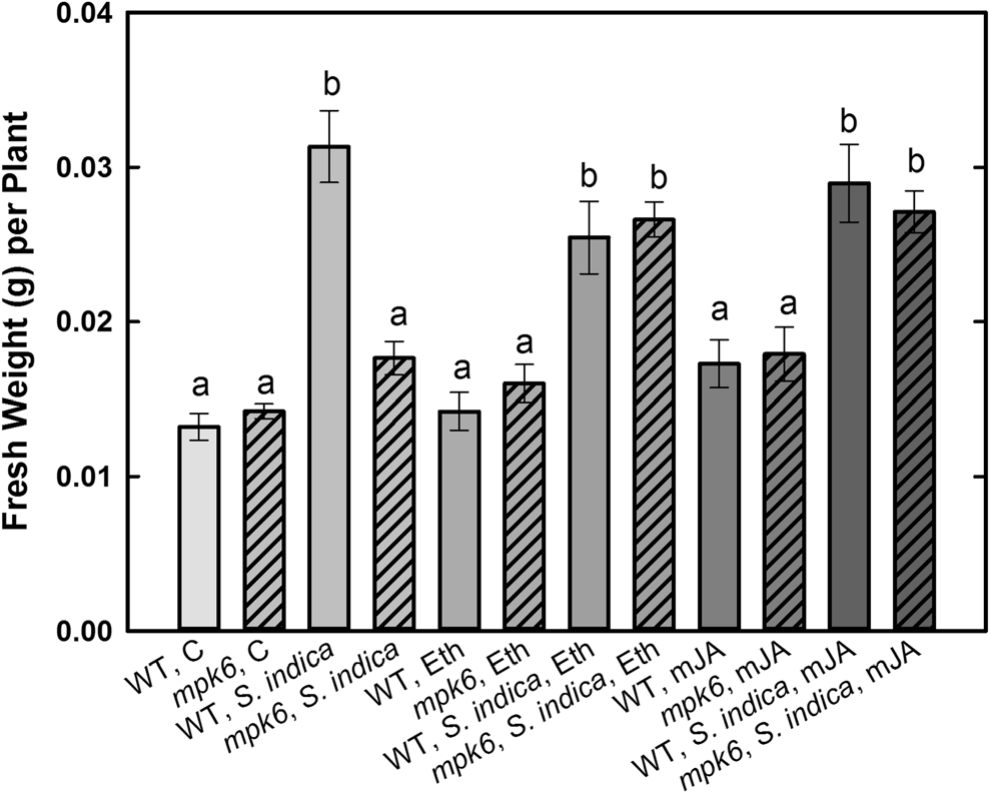
*S. indica*-mediated increase in the fresh weight of wild-type (WT) and *mpk6* plants with and without mJA or Eth treatment at 7 dai. Letters indicate significant differences (*P* < 0.05). Values are means ±SE, *n* = 3

It is known that wild type *A. thaliana* colonized by *S. indica* have higher shoot biomass than the un-colonized control plants (Varma et al. 1999). In contrast, for *mpk6* it was demonstrated that it lacks this typical *S. indica*-triggered effect (Vadassery et al. 2009). Here, we confirm this phenomenon showing no clear growth promotion in *mpk6* plants (Fig. 1). In addition, we tested whether foliar application of ethephone (Eth) or methyl-jasmonate (mJA) prior to fungus inoculation on both wild type and *mpk6* could alter these effects either positively or negatively. Therefore, we determined the fresh weight of *S. indica*-colonized and/or hormone-treated wild-type and *mpk6* plants at 7 dai. Results of three independent experiments show that hormone treatments alone did not enhance the biomass of the wild type. In contrast, a clear restoration of fungus-mediated growth response in *mpk6* after Eth or mJA application was observed, which was comparable to the colonized wild-type plants (Fig. 1).

### *S. indica* Root Colonization Is Hampered in *mpk6* but Restored upon Eth or mJA Application

**Fig. 2:**
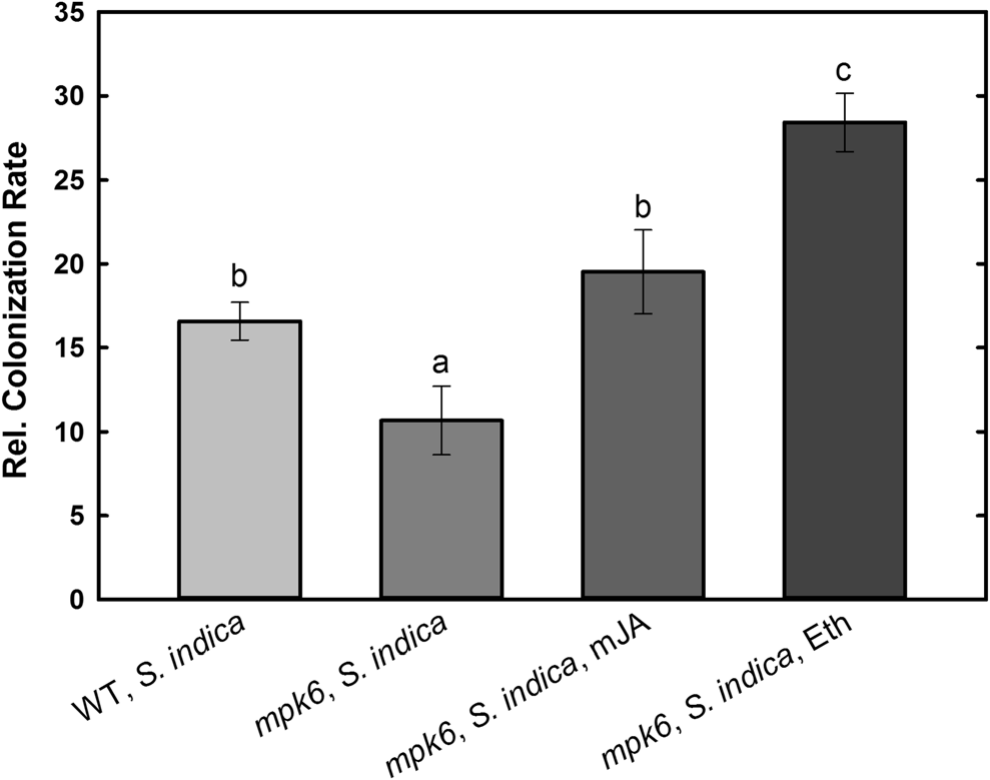
*S. indica* colonization rate in wild-type (WT) and *mpk6* roots at 7 dai. Mutant plants were additionally treated with mJA or Eth. Letters indicate significant differences (*P* < 0.05). Values are means ±SE, *n* = 3

The induction of beneficial effects triggered by *S. indica* in plants depends on proper colonization and further growth of the fungus in the root tissue (Jacobs et al. 2011). Therefore, in the next step we aimed at analyzing the root colonization rate of the fungus in wild-type and *mpk6* roots in our system. The results show that the colonization rate of *mpk6* at 7 dai was significantly lower than the one of wild-type plants (Fig. 2). As shown in the previous experiment, the differences in biomass between un-treated and phytohormone-treated wild-type plants were not significant (Fig. 1). Sherameti et al. (2008) put the degree of fungal colonization and the beneficial responses such as increase in biomass into positive correlation. Therefore, we did not determine the colonization rate in mJA- or Eth-treated wild-type roots. The biomass of phytohormone-treated and colonized *mpk6* plants was significantly higher. Hence, we checked whether Eth or mJA could restore the ability of *S. indica* to colonize the *mpk6* roots properly. Indeed, the results show that *mpk6* plants, which were systemically treated with either phytohormones, could be colonized as efficiently (mJA) or significantly more (Eth) than wild-type roots (Fig. 2).

### Expression of Several Defense- and Hormone-Related Genes in *mpk6* Is Changed upon *S. indica* Colonization and Application of Eth or mJA

**Fig. 3:**
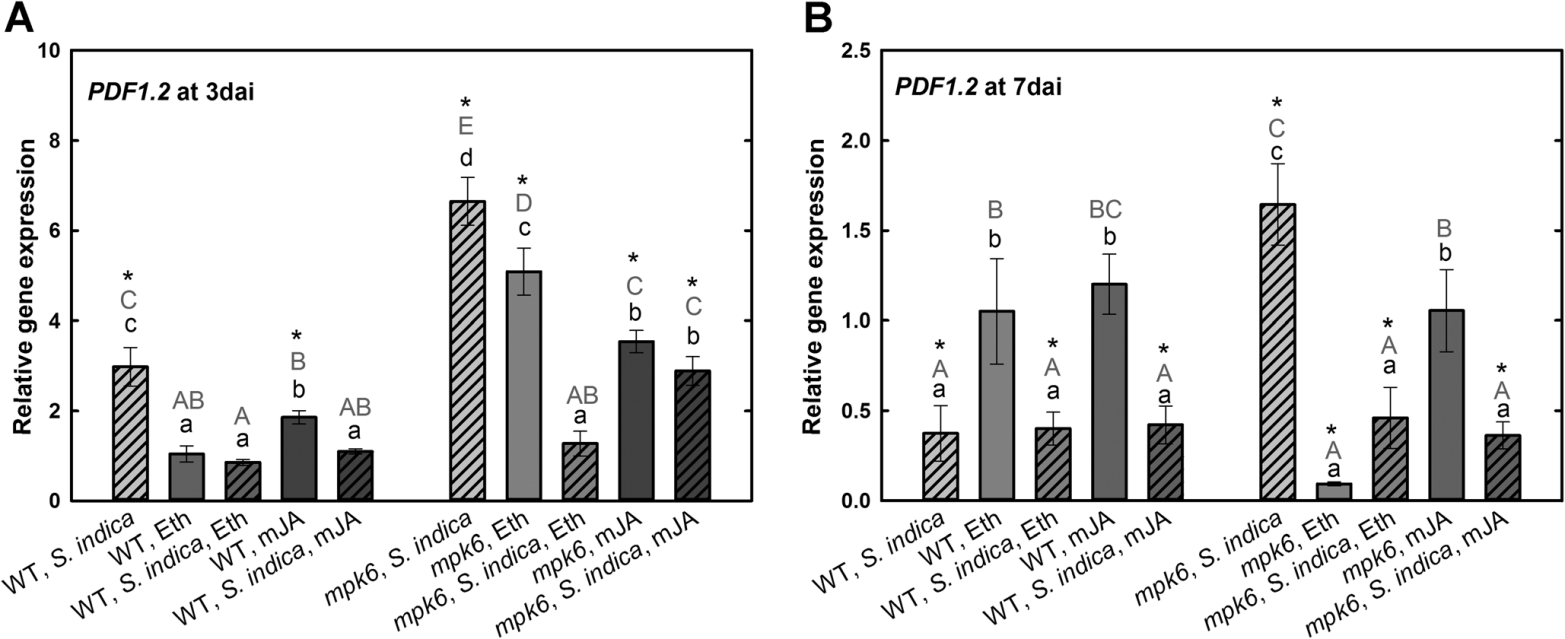
Expression levels of *PDF1.2* in *S. indica*-colonized and -non-colonized wild-type and *mpk6* roots at 3 dai (**a**) and 7 dai (**b**) relative to un-colonized control roots. All plants were additionally treated with mJA or Eth. Values are means ±SE, *n* = 3. Asterisks indicate significant up- or downregulation (*P < 0.05). Lowercase letters indicate significant differences in one group (wild type or *mpk6*) and uppercase letters indicate significant differences between two groups (wild type and *mpk6*)

As previously reported, *S. indica* efficiently suppresses plant immune responses during the biotrophic phase of the root colonization and different components of plant defense and hormone signaling are involved in this process (Jacobs et al. 2011). Therefore, we assessed whether reduced fungal colonization of *mpk6* mutant (Fig. 2) is associated with an impaired ability of the fungus to suppress plant defense responses due to hampered hormone- and defense-related pathways. This prompted us to analyze transcriptional expression of several genes, which are involved in the regulation of plant defense. First, we investigated the expression of plant defense marker gene *PDF1.2*, an ET- and JA-responsive defensin, in both non-colonized and colonized wild-type and mutant roots, with or without hormonal treatment, at both biotrophic (3 dai; Fig. 3a) and cell death-associated root colonization phase of *S. indica* (7 dai; Fig. 3b). Our results demonstrate that both hormones significantly reduced the expression of *PDF1.2* in non-colonized wild-type and mutant plants. In colonized wild-type plants *PDF1.2* was upregulated at biotrophic phase. At the same colonization phase in *mpk6* its expression was strongly enhanced (threefold). Interestingly, Eth and mJA treatments led to reduction in the *PDF1.2* expression in both colonized wild-type and mutant plants at 3 dai. Eth reduced the expression of *PDF1.2* to a greater extent than mJA in colonized mutant plants. *PDF1.2* was significantly downregulated in colonized wild-type roots at 7 dai. Interestingly, these plants were unaffected by hormonal treatments. In *mpk6*, *PDF1.2* expression was still upregulated upon fungal colonization. Similar to biotrophic stage, Eth- and mJA-treated mutant showed strong reduction in *PDF1.2* gene expression. This observation gives the assumption that intact ET and JA pathways are crucial for *S. indica* to suppress plant defense for proper root colonization.

**Fig. 4:**
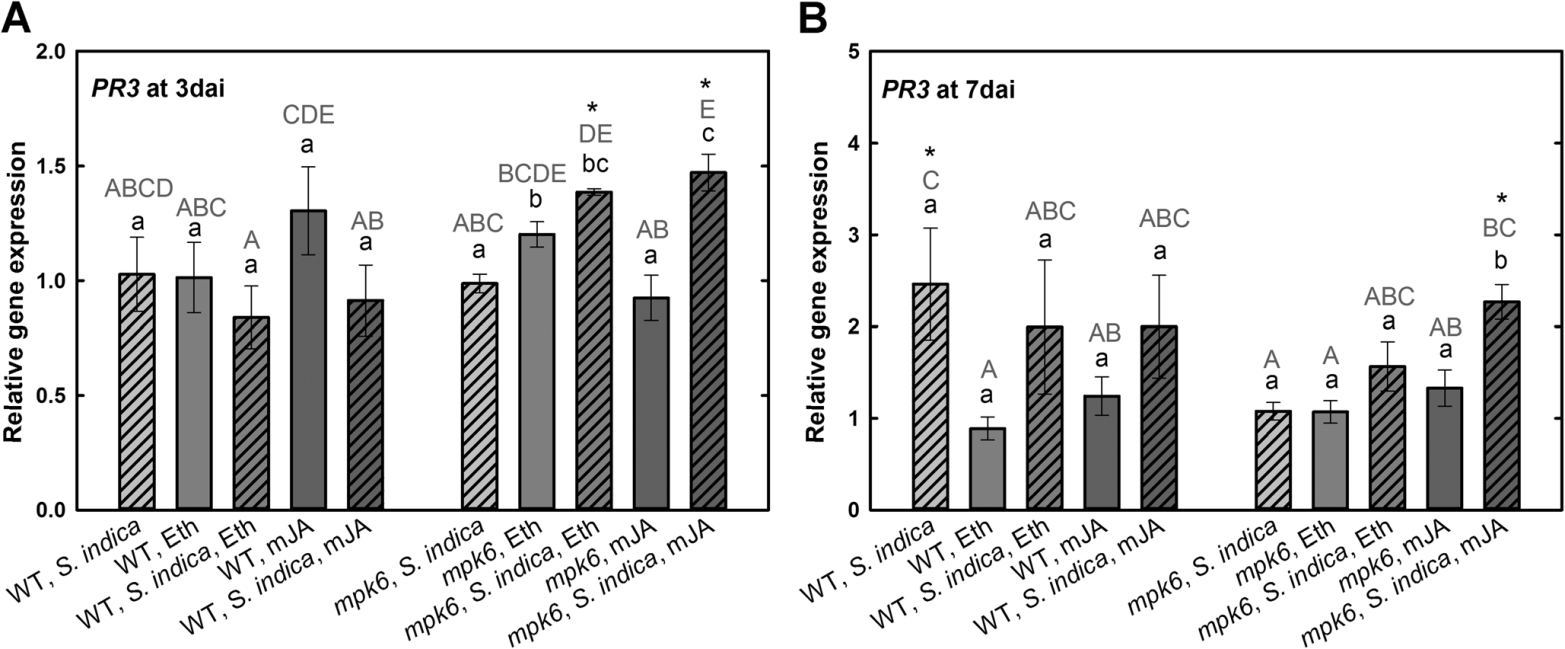
Expression levels of *PR3* in *S. indica*-colonized and -non-colonized wild-type and *mpk6* roots at 3 dai (**a**) and 7 dai (**b**) relative to un-colonized control plants. All plants were additionally treated with mJA or Eth. Values are means ±SE, *n* = 3. Asterisks indicate significant up- or downregulation (*P < 0.05). Lowercase letters indicate differences in one group (wild type or *mpk6*) and uppercase letters indicate differences between two groups (wild type and *mpk6*)

Another pathogenesis-related gene, *PR3*, encoding a basic chitinase, was not regulated in colonized wild-type roots upon any treatment at early colonization phase. Similarly, non-colonized mutant plants did not show any regulation of *PR3* at 3 dai. Only application of Eth or mJA triggered its upregulation. Likewise, in colonized *mpk6* roots Eth or mJA triggered significant upregulation of *PR3* in colonized *mpk6* (Fig. 4a). At 7 dai *PR3* was significantly induced in colonized wild-type plants. Hormones did not have any influence on *PR3* expression in wild-type roots. In colonized *mpk6* mutant plants, however, the expression was significantly elevated only after hormonal treatment, especially upon application of mJA (Fig. 4b).

**Fig. 5:**
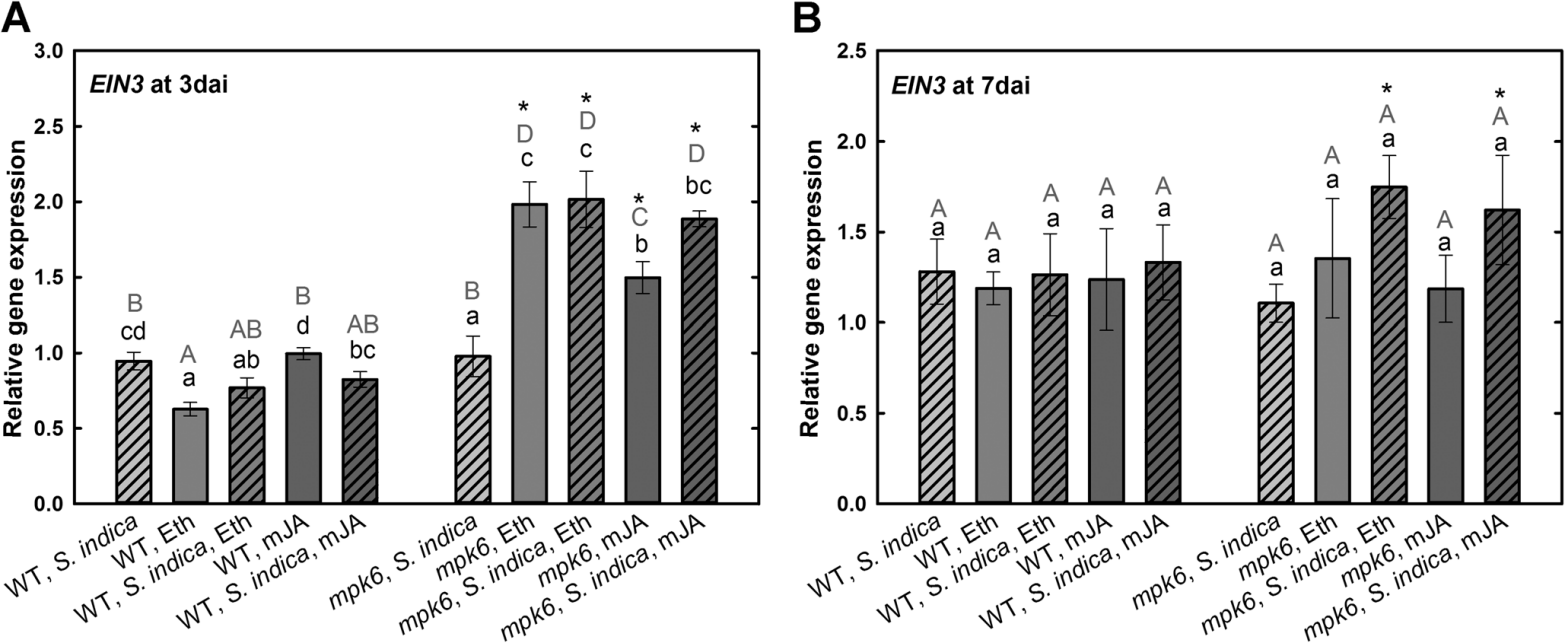
Expression levels of *EIN3* in *S. indica*-colonized and -non-colonized wild-type and *mpk6* roots at 3 dai (**a**) and 7 dai (**b**) relative to un-colonized control plants. All plants were additionally treated with mJA or Eth. Values are means ±SE, *n* = 3. Asterisks indicate significant up- or downregulation (*P < 0.05). Lowercase letters indicate significant differences in one group (wild type or *mpk6*) and uppercase letters indicate significant differences between two groups (wild type and *mpk6*)

**Fig. 6:**
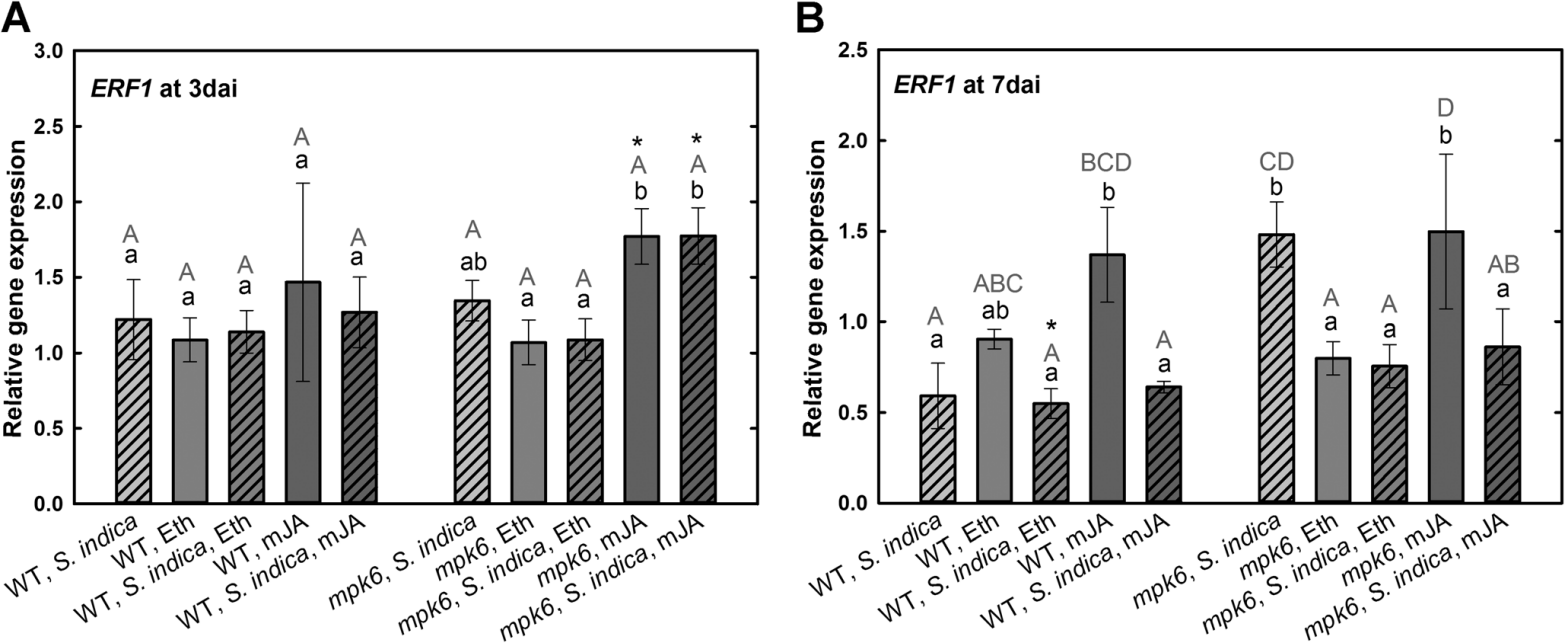
Expression levels of *ERF1* in *S. indica*-colonized and -non-colonized wild-type and *mpk6* roots at 3 dai (**a**) and 7 dai (**b**) relative to un-colonized control plants. All plants were additionally treated with mJA or Eth. Values are means ±SE, *n* = 3. Asterisks indicate significant up- or downregulation (*P < 0.05). Lowercase letters indicate differences in one group (wild type or *mpk6*) and uppercase letters indicate differences between two groups (wild type and *mpk6*)

The nuclear protein EIN3 is a transcription factor in ET signaling pathway that regulates the expression of its immediate target gene ET-responsive factor1, ERF1 (Chao et al. 1997). In *A. thaliana*, besides ET, also JA signaling targets ERF1. It is known that ERF1 functions as a putative activator of PDF1.2 in the beneficial interaction between *S. indica* and *A. thaliana* (Camehl et al. 2010). Our results demonstrate that in wild-type plants at 3 dai *EIN3* is not differentially expressed neither upon *S. indica* colonization nor after mJA treatment. Only Eth treatment led to significant decrease in its expression. In *mpk6*, both hormones, regardless of fungal colonization, significantly induced the expression of *EIN3* (Fig. 5a). Similar to 3 dai, at 7 dai, no change in the expression of this gene in colonized wild-type roots could be observed. Hormones showed no effects on *EIN3* expression in wild-type roots, only Eth or mJA treatments triggered the significant upregulation of *EIN3* in colonized *mpk6* roots (Fig. 5b). Our results obtained with the wild type are in line with the microarray data recently presented by Lahrmann et al. (2015). At 3 dai in wild-type and *mpk6* plants, the expression of *ERF1* was not regulated neither upon *S. indica* colonization nor after hormonal treatments (Fig. 6a). Interestingly, its expression was increased only after mJA treatment in both non-colonized and colonized mutant roots. At 7 dai, *ERF1* was slightly but not significantly upregulated upon mJA treatment in wild-type roots. Otherwise it was down-regulated in *S. indica*-colonized wild-type roots after hormonal treatments. At 7 dai it was significantly up-regulated in colonized *mpk6* plants compared with wild type. Treatment with either hormones, however, decreased the expression level of *ERF1* in colonized mutants (Fig. 6b).

**Fig. 7:**
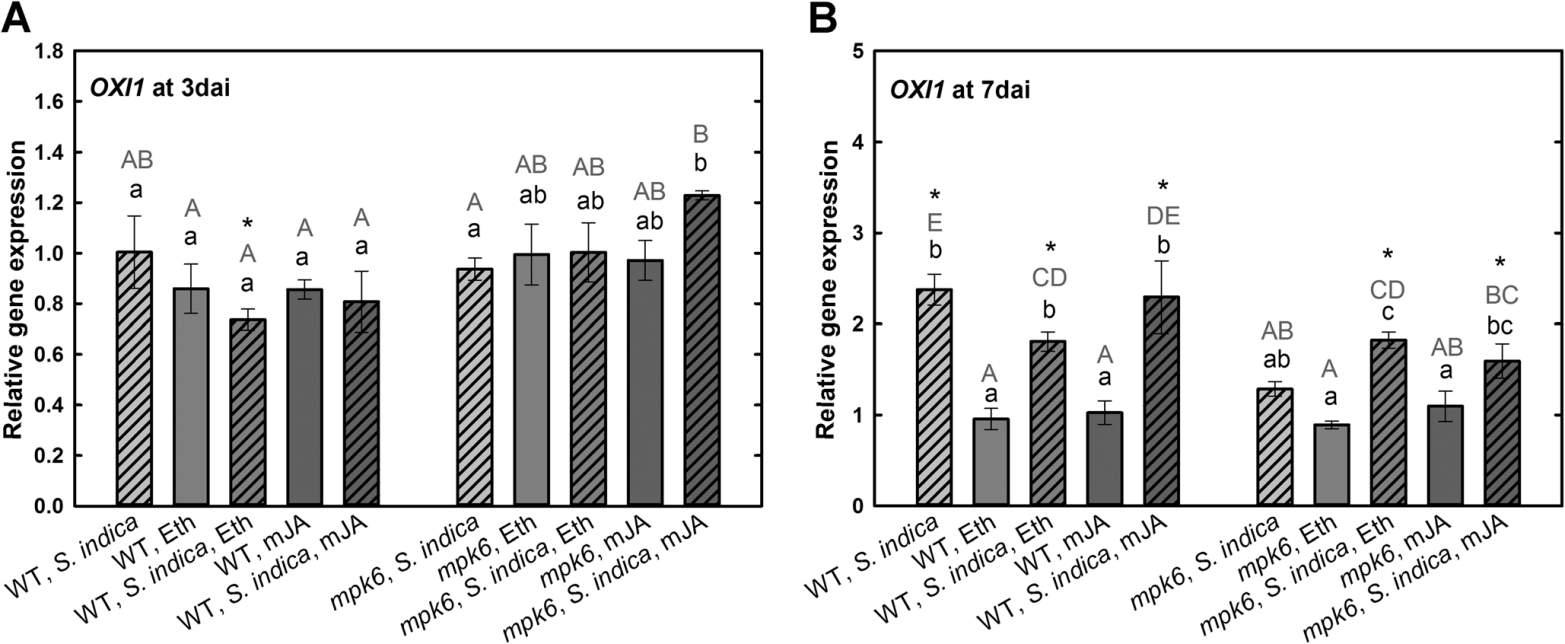
Expression levels of *OXI1* in *S. indica*-colonized and -non-colonized wild-type and *mpk6* roots at 3 dai (**a**) and 7 dai (**b**) relative to un-colonized control plants. All plants were additionally treated with mJA or Eth. Values are means ±SE, *n* = 3. Asterisks indicate significant up- or downregulation (*P < 0.05). Lowercase letters indicate differences in one group (wild type or *mpk6*) and uppercase letters indicate differences between two groups (wild type and *mpk6*)

The serine/threonine kinase protein oxidative signal inducible 1 (OXI1) belonging to the AGC protein kinase family is required for full activation of MPK6 (Rentel et al. 2004). *OXI1* is one of the genes responsible for the typical growth phenotype induced by *S. indica* (Camehl et al. 2011). Our results show that the transcript level of *OXI1* was not changed in colonized wild-type roots at biotrophic phase. The expression of *OXI1* was also not changed in *mpk6* roots upon *S. indica* colonization at 3 dai, however, it was significantly upregulated in colonized *mpk6* roots after application of mJA compared to noncolonized mutants (Fig. 7a). At 7 dai, the expression of *OXI1* was significantly induced upon *S. indica* colonization in wild-type plants (Fig. 7b). Treatment with hormones showed no effect on its expression in wild-type roots. Upon hormonal treatments, significant upregulation of *OXI1* in *S. indica*-colonized wild-type plants was observed. In colonized *mpk6*, no change in *OXI1* expression could be detected. Neither of the hormones triggered any changes in its expression in non-colonized *mpk6* roots. In colonized roots, however, Eth or mJA increased significantly the expression level of *OXI1*.

**Fig. 8:**
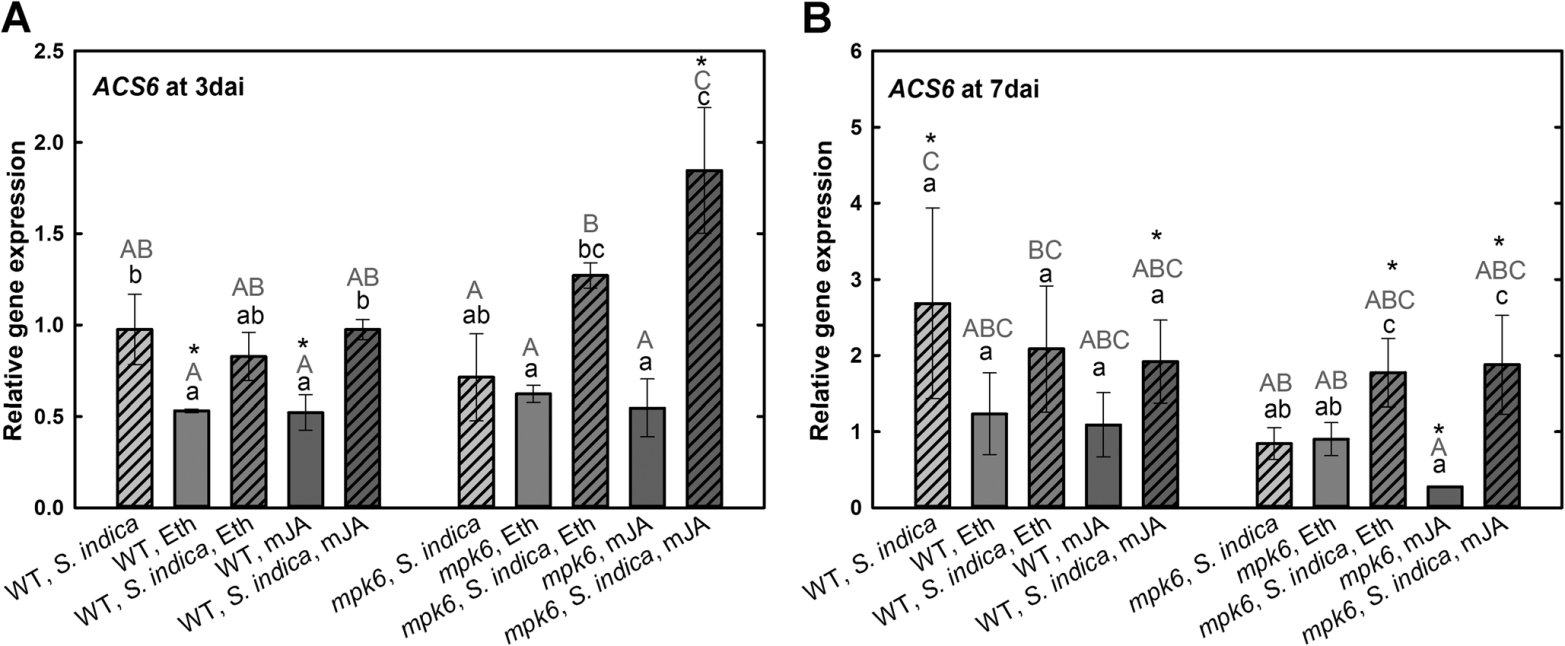
Expression levels of *ACS6* in *S. indica*-colonized and -non-colonized wild-type and *mpk6* roots at 3 dai (**a**) and 7 dai (**b**) relative to un-colonized control plants. All plants were additionally treated with mJA or Eth. Values are means ±SE, *n* = 3. Asterisks indicate significant up- or downregulation (*P < 0.05). Lowercase letters indicate differences in one group (wild type or *mpk6*) and uppercase letters indicate differences between two groups (wild type and *mpk6*)

*ACS6* encodes an 1-aminocyclopropane-1-carboxylate ACC synthase (ACS), which is the rate limiting enzyme in ET synthesis (Tsuchisaka and Theologis 2004). At 3 dai, wild-type plants did not show any changes in transcription level of *ACS6*. Similarly, there was no regulation of this gene in colonized wild-type plants upon either of the hormonal treatments. However, in non-colonized wild-type roots Eth or mJA applications led to significant reduction of *ACS6* expression. In *mpk6* at 3 dai transcription level was reduced upon application of either hormones. In colonized *mpk6* treatments with Eth or mJA significantly increased the expression of *ACS6* (Fig. 8a). At 7 dai, its expression was induced in all colonized wild-type roots with or without hormonal application. In *S. indica*-colonized *mpk6* roots, the *ACS6* expression was elevated upon treatments with Eth and mJA (Fig. 8b).

## Discussion

In plants, pathogen- or microbe-associated molecular patterns (PAMPs and MAMPs) are recognized by a set of pattern recognition receptors (PRRs). PRRs trigger than various responses such oxidative burst, activation of mitogen-activated protein kinases (MAPKs) and transcriptional reprogramming, often leading in case of pathogen attack to PAMP-triggered immunity (PTI). Stress-activated MAPKs as central components of subsequent cell signaling cascades transduce extracellular signals into a set of different plant defense responses (Cazole et al. 1999; Kiegerl et al. 2000; Asai et al. 2002; Doczi et al. 2007; Meng and Zhang 2013). They are positive regulators of plant defense, in which different plant hormones such as jasmonic acid (JA) and ethylene (ET) play a crucial role (Bethke et al. 2009). For instance, MPK3 and MPK6, together with JA, are essential for plant defense against *Botrytis cinerea* as *mpk3* (Ren et al. 2008) and *mpk6* (Méndez-Bravo et al. 2011) mutants, as well as lines with attenuated MAPK activities were affected in defense against this fungal necrotroph (Schweighofer et al. 2007). This emphasizes the crucial role of MAPKs in plant–pathogen interactions (Han et al. 2010; Galletti et al. 2011; Tena et al. 2011; Meng and Zhang 2013). Interestingly, these early plant reactions occurring during pathogenic plant-microbe interactions are fairly similar to those taking place during symbiotic relationships (Parniske 2000; Herouart et al. 2002; Parniske 2004). Hence, it has been proposed that similar basic defense events are activated during early phases of plant colonization by symbiotic organisms (Carden and Felle 2003). For instance, it was recently suggested that MAPKs might regulate the response of symbiosis between soybean and arbuscular myccorrhizal fungus (Liu et al. 2015). The activation of MAPK cascades and associated signaling pathways seem to be similarly important during symbiosis between plants and sebacinoid endophytic fungi (Vadassery et al. 2009). Accordingly, here we present new evidences showing that MPK6 indeed plays a crucial role during the beneficial interaction between *S. indica* and *A. thaliana*. Previously, Vadassery et al. (2009) showed that the cell wall extract from *S. indica* triggers the activation of MPK6 and the *mpk6* mutant failed to respond with typical growth promotion to the fungus root colonization. We confirmed this finding and show here that it might be due to the lower colonization rate of *mpk6* when compared to the wild type. The normal colonization rate could be, however, restored upon application of phytohormones Eth or mJA. Thus, treatment with mJA increases the colonization rate in *mpk6* resembling its level in the wild-type roots, while the application of Eth leads to its significant elevation beyond the wild-type level. What might be the reasons for that? Most probably, it is because the plant is not able to control the colonization process of the fungus due to the lack of MPK6. This might be interlinked with the compromised general plant immune system in *mpk6*, which, in compatible interaction, is required to keep the fungal colonization and endophytism balanced (Fese and Zuccaro 2016). Further, this kinase regulates e.g., ACS6, which is the rate limiting enzyme in ET synthesis (Tsuchisaka and Theologis 2004) modulating ET production (Liu and Zhang 2004). Hence, the knock-out of MPK6 might lead to lower ET levels in *mpk6* mutant as previously suggested by Xu et al. (2008). This is supported by the fact that in the *mpk6* mutant the ET level decreased only slightly upon infection with *B. cinerea* (Han et al. 2010). In wild-type scenario at the onset of the colonization process *S. indica* is confronted with a functional plant immune system and the fungus does not evade host detection but rather suppresses immunity by various MAMPs (Jacobs et al. 2011) or specific effectors. After the establishment of a stable beneficial interaction, almost no defense or stress genes are activated and no reactive oxygen species (ROS) are produced by the host against *S. indica* (Vahabi et al. 2015). Similarly, Jacobs et al. (2011) demonstrated that *S. indica* counteracts the immune system as indicated by the lack of oxidative burst and MAMP–induced reduction of seedling growth. It is suggested that, at least partially, JA signaling during this process contributes to the suppression of immunity (Jacobs et al. 2011). Our data are in accordance with results presented by Jacobs et al. (2011) demonstrating reduced *S. indica* root colonization at 7 dai in JA signaling mutant, *jasmonate insensitive1-1* (*jin1-1*) and the JA biosynthesis mutant *jasmonate resistant1-1* (*jar1-1*). The authors explain the reduced colonization of both mutants with an elevated immune-related gene expression, which determines the significance of intact JA signaling pathway for symbiosis. For ET it is proposed that *S. indica* induces its synthesis in barley and *A. thaliana* roots during root colonization (Khatabi et al. 2012). Consequently, impaired ET signaling leads to reduced root colonization, whereas mutants with constitutive ET signaling are hyper-susceptible to the fungus (Khatabi et al. 2012). Further, our results are in line with Khatabi et al. (2012), who showed higher colonization rate of *S. indica* in mutants that displayed constitutive ethylene signaling (*ctr1-1*) or enhanced ethylene synthesis (*eto1-1*) during cell death-associated phase (7 dai).

Beside of the changes in phytohormone levels, expression of a broad set of genes regulated by MAPK-mediated signaling pathways (Colcombet and Hirt 2008) is impaired in *S. indica*-colonized roots (Lahrmann et al. 2015). Here, we demonstrate that altered expression patterns of several defense and signaling genes controlled by MPK6 hamper on the one hand, proper root colonization and on the other hand, the development of typical growth promotion triggered by *S. indica*. We show that *S. indica* does not suppress the expression of plant defense genes such as *PDF1.2* and *ERF1* in *mpk6* roots, which could be, however, partially restored upon application of mJA or Eth. Induction of *PDF1.2* depends on synergistic action of both ET and JA signaling pathways, which are regulated by MPK6 (Kunkel and Brooks 2002). Our results show that *PDF1.2* is highly upregulated in *mpk6* during biotrophic colonization phase of *S. indica*. Foliar application of both mJA or Eth decreased expression of this defensin to the similar level measured in wild-type roots (Eth) or even lower (mJA). In *mpk6* at cell death-associated phase (7 dai) we observed still significant upregulation of *PDF1.2*, which was again significantly suppressed upon application of both hormones.

The expression of *PDF1.2* is regulated by ERF1, an ethylene transcriptional activator (ethylene response factor 1) (Camehl et al. 2010). It is modulated by the transcription factor EIN3 (ethylene insensitive 3), which generally positively regulates gene expression in response to ET (Chao et al. 1997). Our results demonstrate that although the transcription level of *EIN3* at biotrophic colonization phase (3 dai) in *mpk6* is comparable to wild-type level, it is significantly elevated upon hormone application, especially after Eth treatment. In *mpk6* at cell death-associated phase (7 dai), Eth application led to an increase in *EIN3* expression. Interestingly, previous studies revealed that levels of *EIN3* mRNA and protein in wild-type plants are unaffected after ET treatment (Chao et al. 1997), which is in accordance with our results. Further, it was showed that a MAPK kinase cascade is involved in the ET signaling pathway (Kieber and Ecker 1993). Thus, as the level of *EIN3* mRNA is increased in colonized *mpk6* plants after application of exogenous Eth at both colonization phases, it is possible that Eth compensates the impaired MAPK signaling by *EIN3* upregulation, which in turn supports the *S. indica* colonization. It has also been shown that ET regulates EIN3 via inhibition of EIN3 protein degradation (Guo and Ecker 2003). Hence, to make final statements it would be necessary to check the levels of EIN3 protein in roots colonized by *S. indica*. In the case of ERF1, which is the target of EIN3 in ethylene signaling pathway and plays a central role in ethylene-associated defense signaling in *A. thaliana* (Camehl et al. 2010), at both colonization phases, neither of the hormones had any influence on its expression in the wild type. We show, however, a significant upregulation of this gene upon mJA treatment in colonized *mpk6* roots at 3 dai. Interestingly, at 7 dai, Eth or mJA treatments reduce significantly *ERF1* expression in *mpk6*. This shows that these two plant hormones have different effects on regulation of *ERF1* expression. We conclude that in *mpk6*, due to not properly functioning hormone signaling pathways, the application of mJA might lead to an upregulation of *ERF1*, which might contribute to root colonization similar to the wild-type level. This is in agreement with the results of Khatabi et al. (2012), who demonstrated higher colonization rate in *ERF1* overexpressing line. Further, Eth application causes reduction of *ERF1* expression, which in turn results in decrease in *PDF1.2* expression, which can again support the colonization process and maintain the symbiosis.

For the full activation of MPK6 an *oxidative signal inducible* (OXI1) serine-threonine kinase is required after e.g., treatment with pathogen-derived elicitors (Rentel et al. 2004). Camehl et al. (2011) showed that the upregulation of OXI1 is an important factor for appearance of *S. indica* beneficial effects. Our results are in agreement with those data, which show a significant upregulation of *OXI1* at 7 dai in wild-type roots. However, this was not the case in colonized *mpk6* roots at 7 dai. The increase in *OXI1* expression in *mpk6* in response to *S. indica* colonization could be only observed when the plants were treated with Eth or mJA. This suggests the involvement of both hormones and MPK6 in *OXI1* pathway. The fact that OXI is phosphorylated by the MPK6 in vitro, which might result in MPK6-OXI1 feedback loop (Forzani et al. 2011) strengthens this assumption.

Pathogenesis-related PR-3, which encodes a basic chitinase, is activated against necrotrophic fungi primarily by the ET/JA pathway (Van Wees et al. 2008). Recently, Vahabi et al. (2015) showed upregulation of several different *A. thaliana PR* genes two and 6 days after co-cultivation with *S. indica*. Here, although hormone-treated wild-type plants did not show any changes in *PR-3* expression at biotrophic colonization phase (3 dai), the *mpk6* exhibited significant upregulation of *PR-3* after Eth or mJA application. At cell death-associated phase (7 dai), *PR-3* was upregulated in colonized wild-type roots and in *mpk6* mutant roots only upon mJA application. Upregulation of *PR-3* at 7 dai in the wild-type roots suggests a different function of this gene in the interaction between plants and *S. indica*. This is supported by recent report suggesting that PR proteins have various functions and they are also involved in different processes other than defense (Delaunois et al. 2013).

As mentioned above, MPK6 is required for ethylene induction in *A. thaliana* and two isoforms of 1-aminocyclopropane-1-carboxylic acid synthase (ACS), ACS2 and ACS6, are substrates of MPK6. Phosphorylation of ACS2 and ACS6 by MPK6 triggers elevated ACS activity resulting in higher ethylene production (Liu and Zhang 2004), which supports the root colonization of *S. indica* (Khatabi et al. 2012). Our results show that the application of Eth or mJA does not cause elevation in *ACS6* expression during the biotrophic colonization phase of *S. indica* in wild-type plants. However, higher expression of *ACS6* in *mpk6* upon Eth or mJA treatment at early colonization phase (3 dai) supports our hypothesis that hampered hormone biosynthesis and signaling might be the reason for the lack of the growth promotion triggered by *S. indica* in these plants. At cell death-associated phase (7 dai), the expression of *ACS6* reaches its maximum in un-treated wild type. This is in line with findings presented by Khatabi et al. (2012), who demonstrated that *S. indica* colonization promotes ET biosynthesis, which was not the case in *mpk6* as shown here. However, application of plant hormones led to a significant elevation of *ACS6* expression in colonized mutant plants. According to Khatabi et al. (2012), when ethylene signaling is indeed saturated, treatment with ACC, the immediate precursor of ET, would not further affect ET synthesis and thus fungal root colonization. This is in agreement with our results showing unchanged *ACS6* expression in wild-type plants upon application of Eth. In *mpk6* plants with the improper ET signaling, however, the application of Eth led to a significant increase in *ACS6* expression, which again supports fungal colonization in this mutant.

Our results demonstrate that successful *S. indica* colonization in *A. thaliana* root in large part depends on efficient suppression of plant immune responses, which relies on intact hormone signaling. Our conclusions are based on extended transcriptional studies. Given the fact that some of the encoded proteins are not regulated at the transcriptional level, in the deepening studies in the future experiments on the protein level should be carried out to strengthen our arguments. But irrespective of that, here we clearly show that the lack of MPK6 leads to absence of the typical growth promotion effects usually seen in the colonized wild-type plants but also has a negative impact on the root colonization process itself. Foliar Eth or mJA application as well as expression analysis of several defense- and hormone-related genes in the wild type and *mpk6* strongly support our conclusions. The possible crosstalk with other pathways e.g., salicylic acid, however, which might be primary targets for MPK6 as well as involvement of other kinases (e.g., MPK3) should be analyzed in further experiments.

## Materials and Methods

### Growth Conditions of *A. thaliana* and Fungus

Seeds of both *A. thaliana* (Col-0) and *mpk6-2* (SALK_073907) mutant were surface-sterilized and placed on Petri dishes containing Murashige and Skoog nutrient medium. After cold treatment at 4 °C for 48 h, plates were incubated for 9 days in a 16 h light/8 h dark (L16D8) photoperiod at 23 °C. *S. indica* was cultured as described previously (Varma et al. 1999).

### Co-cultivation Experiments, Hormone Treatment and Determination of Fresh Weight

Nine-day-old seedlings were transferred to a modified MMN culture medium (MMN_1/10_ medium with a 1/10 ratio of nitrogen and phosphorus and no carbohydrate). Always two seedlings were placed on top of a 90-mm Petri dishes containing culture medium. After 24 h, hormones were applied to the shoots of 10-day-old plants under sterile conditions in two droplets onto two leaves per seedling in following concentrations: 60 nM methyl-jasmonate (mJA; Sigma-Aldrich), 400 nM ethephone (Eth; Sigma-Aldrich). This final selected concentration did not result in any phenotypical changes (data not shown). After 24 h, fungal plugs of 5 mm in diameter were placed at a distance of 1 cm from the roots. Plates were incubated subsequently in growing chamber till the corresponding analysis. For determination of fresh weight, the seedlings were harvested at 7 dai*.*

### Gene Expression

For both quantification of fungal colonization and the gene expression analysis whole roots of approximately 15–20 *A. thaliana* (Col-0) or *mpk6* mutant seedlings were sampled. RNA was extracted using Qiagen RNA Plant Mini Kit according to the manufacturer’s instructions (Qiagen, Hilden, Germany). RNA was analyzed using a Nanodrop 2000c Spectrophotometer (Thermo Scientific, Peqlab, Germany). cDNA synthesis was performed using SuperScriptIII reverse transcriptase (Invitrogen, Carlsbad, CA, USA) according to the manufacturer’s instructions. For fungal colonization analysis *PiTef1* was used. For all experiments as reference gene *UBQ5* was used. For primer sequences see Table 1. qPCR was performed using ABI PRISM 7300 (Applied BioSystems, Waltham, MA, USA). The final reaction volume was 25 μl. Samples were analyzed in three biological and three technical replicates. At the end of each PCR run, a dissociation curve was added to rule out unspecific reactions or primer dimmers. The PCR reaction was conducted as follows: 95 °C for 10 min, then 40 cycles of 95 °C for 15 s and 60 °C for 60 s. Changes in transcript levels were calculated using the 2^-∆∆ct^ method (Schmittgen and Livak 2008).

**Table 1:** List of primers for qPCR used in this study

ERF1 reverse	TCCCACTATTTTCAGAAGACCCC
ERF1 forward	CGGCGGAGAGAGTTCAAGAGTC
EIN3 reverse	AGCTTGTGGAACAGGAC
EIN3 forward	CATTTCTCCAGGTTACAATGAT
PR3 reverse	TGCTGTAGCCCATCCACCTG
PR3 forward	ATCACCGCTGCAAAGTCCTTC
OXI1 reverse	CGTCGCTCCATACAACATCT
OXI1 forward	TCATCTACATTGGCCGTGTC
PDF1.2 reverse	ACCCCTGACCATGTCCCACTTGG
PDF1.2 forward	CTGCTTTCGACGCACCGGCAA
ASC6 reverse	CGGTCTTAAGTCTGTGCACGG
ASC6 forward	CCGGGAATGTTTGAAGTCTCTTG
Pitef1 reverse	TCGTCGCTGTCAACAAGATG
Pitef1 forward	ACCGTCTTGGGGTTGTATCC
UBQ5 reverse	ATGACTCGCCATGAAAGTCC
UBQ5 forward	CCAAGCCGAAGAAGATCAAG

### Statistics

All data are obtained at least from three independent biological replicates and differences are analyzed by one-way ANOVA with a LSD post hoc test. Statistical analysis was conducted using StatGraphics plus 4.0 software (Statpoint Technologies Inc., Warrenton, VA, USA). The data are checked for homogeneity of variance and *P* < 0.05 was used to determine significance.
